# Functional and effective connectivity analysis of drug-resistant epilepsy: a resting-state fMRI analysis

**DOI:** 10.3389/fnins.2023.1163111

**Published:** 2023-04-20

**Authors:** Eric Jacob Bacon, Chaoyang Jin, Dianning He, Shuaishuai Hu, Lanbo Wang, Han Li, Shouliang Qi

**Affiliations:** ^1^College of Medicine and Biological Information Engineering, Northeastern University, Shenyang, China; ^2^Key Laboratory of Intelligent Computing in Medical Image, Ministry of Education, Northeastern University, Shenyang, China; ^3^Department of Neurosurgery, Shengjing Hospital of China Medical University, Shenyang, China; ^4^Department of Radiology, Shengjing Hospital of China Medical University, Shenyang, China

**Keywords:** default mode network, dorsal attention network, resting-state functional magnetic resonance, functional connectivity, effective connectivity, epilepsy

## Abstract

**Objective:**

Epilepsy is considered as a neural network disorder. Seizure activity in epilepsy may disturb brain networks and damage brain functions. We propose using resting-state functional magnetic resonance imaging (rs-fMRI) data to characterize connectivity patterns in drug-resistant epilepsy.

**Methods:**

This study enrolled 47 participants, including 28 with drug-resistant epilepsy and 19 healthy controls. Functional and effective connectivity was employed to assess drug-resistant epilepsy patients within resting state networks. The resting state functional connectivity (FC) analysis was performed to assess connectivity between each patient and healthy controls within the default mode network (DMN) and the dorsal attention network (DAN). In addition, dynamic causal modeling was used to compute effective connectivity (EC). Finally, a statistical analysis was performed to evaluate our findings.

**Results:**

The FC analysis revealed significant connectivity changes in patients giving 64.3% (18/28) and 78.6% (22/28) for DMN and DAN, respectively. Statistical analysis of FC was significant between the medial prefrontal cortex, posterior cingulate cortex, and bilateral inferior parietal cortex for DMN. For DAN, it was significant between the left and the right intraparietal sulcus and the frontal eye field. For the DMN, the patient group showed significant EC connectivity in the right inferior parietal cortex and the medial prefrontal cortex for the DMN. There was also bilateral connectivity between the medial prefrontal cortex and the posterior cingulate cortex, as well as between the left and right inferior parietal cortex. For DAN, patients showed significant connectivity in the right frontal eye field and the right intraparietal sulcus. Bilateral connectivity was also found between the left frontal eye field and the left intraparietal sulcus, as well as between the right frontal eye field and the right intraparietal sulcus. The statistical analysis of the EC revealed a significant result in the medial prefrontal cortex and the right intraparietal cortex for the DMN. The DAN was found significant in the left frontal eye field, as well as the left and right intraparietal sulcus.

**Conclusion:**

Our results provide preliminary evidence to support that the combination of functional and effective connectivity analysis of rs-fMRI can aid in diagnosing epilepsy in the DMN and DAN networks.

## 1. Introduction

Epilepsy is a common and serious neurological disorder that affects around 50 million people worldwide (World Health Organization [WHO], 2023). Drug-resistant epilepsy (DRE) also known as refractory epilepsy is one of the most complex types of epilepsy. DRE is a complicated and heterogeneous condition in which seizures persist despite adequate trials of two or more carefully chosen and used antiepileptic drugs (AEDs). The term “adequate trial” refers to the use of AEDs at therapeutic doses for a sufficient period of time. The International League Against Epilepsy (ILAE) defines adequate trial failure as the persistence of disabling seizures despite the use of two tolerated, appropriately chosen and used AED schedules, either alone or in combination (Scheffer et al., 2017). Around 20%–30% of the epileptic population remains refractory to treatment and is considered to have drug-resistant epilepsy (Dalic and Cook, 2016).

Diagnosis of DRE has remained challenging for neuroscientists and researchers. This difficulty can be explained by a variety of factors, including a lack of consensus definition, complex seizure semiology, limited diagnostic tools, comorbidities, and medication adherence issues (Jehi et al., 2022). For accurate diagnosis and management, a multidisciplinary approach and careful consideration of the patient’s individual circumstances are required. Treatment options that can help mitigate these challenges include optimizing AED therapy, surgical intervention, non-surgical interventions, complementary and alternative medicine, multidisciplinary care, and education and support. Recent technological advancements have simplified the process by providing numerous credible methods for analyzing and diagnosing brain diseases.

The development of new technology in neuroimaging has brought new insights into how brain disease can be diagnosed and cured. Many technologies for observing brain function non-invasively were developed and used to acquire brain signals. Among them, functional magnetic resonance imaging (fMRI) is considered one of the most prominent in the field (Glover, 2011). This technology enabled the observation of regional brain activation by detecting the amount of oxygen in blood in each part of the brain. Here, we assume that the active region consumes more oxygen for energy. Resting state and task-based are two main kinds of fMRI utilized in the neuroimaging study. The main difference between the two lies in the acquisition procedure. Task-based fMRI is acquired when the subject performs specific tasks. In contrast, a resting state is acquired when the subject rests (Logothetis, 2008).

Understanding the properties of the brain network may help guide surgical intervention for better postoperative outcomes in drug-resistant epilepsy. A variety of resting state networks, each showing a definite spatial topography and putatively corresponding to a specific brain function (Yun et al., 2022). One of them, known as the default-mode network (DMN), is the most famous and essential network for the resting condition, as it consistently shows increased activity during rest than during active and passive cognitive tasks (Yun et al., 2022). The DMN areas typically comprise the posterior cingulate (PCC), medial prefrontal cortex (MPFC), and inferior parietal cortex (IPC) (Raichle et al., 2015). Same as DMN, the dorsal attention network (DAN) is another crucial resting-state fMRI network known to be active when performing specific tasks. However, DAN has also been proven to be associated with mesial temporal epilepsy in a resting state (Szczepanski et al., 2013). The DAN is centered on bilateral regions of the frontal and parietal cortex, including the frontal eye field (FEF) and the intraparietal sulcus (IPS).

Analyzing resting state fMRI (rs-fMRI) data remains challenging for neuroscientists. Brain connectivity modeling is a well-studied approach to illustrating brain function. One of the popular fMRI methods for studying brain networks is functional connectivity (rs-FC). Rs-FC measurements can detect coherent spontaneous neuronal activities within a brain network (van den Heuvel and Hulshoff Pol, 2010; Hlinka et al., 2011). This method has been explored by many researchers and is based on the temporal correlation between BOLD signals (Blood oxygenation level dependency) in distant brain regions. Lee et al. (2014) investigated changes in FC in brain networks for partial refractory epilepsy. The analysis was based on rs-fMRI using intrinsic connectivity contrast (ICC). Liu et al. (2021) also evaluated functional connectivity patterns in epilepsy associated with focal cortical dysplasia (FCD) to explore the underlying pathological mechanism of this disorder. Rs-FC is probably a good analytical approach to investigate rs-fMRI networks, but recent studies have highlighted some weaknesses and limitations of this approach (Buckner et al., 2013). These weaknesses could be covered by other analytical approaches, such as effective connectivity (EC) analysis. This method provides adequate details on directed causal influences between different regions of interest and covers some of the shortcomings observed during the FC analysis. Many studies have supported this using a similar technique to perform brain network assessments and diagnose brain diseases. Using rs-fMRI, Wei et al. (2016) performed an effective connectivity analysis to examine idiopathic generalized epilepsy-related changes in major neurocognitive brain networks. Jiang et al. (2018) also assessed the functional and effective connectivity of the attention and default mode networks of rs-fMRI. Although, the EC method has many advantages and covers some limitations of FC analysis. However, several questions remain unanswered, so relatively few studies characterizing effective or directed connectivity analysis of epileptic disease exist. From all these observations, concrete actions must be taken to create a more efficient and precise method to eliminate these shortcomings.

We propose to analyze functional and effective connectivity based on rs-fMRI in this study. Our study aims to explore brain activities within the DMN and DAN of candidates with DRE. We hypothesize that studying functional and causal interactions within the brain network could help characterize and localize the epileptic zone. We also hypothesize that DRE candidates will have frequent seizures as a result of impaired brain communication caused by abnormal connectivity. Effective and functional connectivity analysis will be performed to achieve our goal. The idea behind such an approach is to effectively contribute to the diagnosis of the epileptogenic zone and facilitate surgical operations by making them more precise.

Our study is organized as follows. The functional connectivity analysis will first be performed using the CONN toolbox to analyze rs-fMRI candidates. It will consist of performing a seed-based correlation analysis to assess the connectivity patterns between each patient and the healthy controls for DMN and DAN separately. Next, the effective connectivity analysis will be performed using the DCM (Dynamic Causal Modeling) approach and the statistical Bayesian modeling inference, including Bayesian model selection and averaging. Finally, we evaluated our findings through statistical analysis. A two-sample *t*-test was conducted to differentiate connectivity within each network for the patients and healthy groups. This original study assesses the applicability of seed-based correlation and DCM analysis based on rs-fMRI data to diagnose epileptic networks.

## 2. Materials and methods

### 2.1. Participants

The dataset in this study initially consisted of 71 subjects (38 patients and 33 HCs). To increase the population size in this study, the data was collected from three different sources (2 samples of patients and a single sample of healthy controls). The first sample consisted of 12 patients who underwent presurgical evaluation from January 2018 to July 2019 at Shengjing Hospital of China Medical University. The evaluation involved a detailed clinical history and neurological examination, complete neuropsychological evaluation, psychiatric assessment, inter-ictal and ictal onset patterns in long-term scalp video-electroencephalogram (video-EEG), magnetic resonance imaging (MRI), and fMRI results. All 12 patients underwent surgical resection for medically refractory epilepsy with histopathological confirmation of FCD.

The others two samples were collected from an open-source website.^1^ The second sample corresponding to the second group of patients is provided by Thompson et al. (2020). Participants were patients with medically drug-resistant who had elected to undergo neurosurgical treatment for their epilepsy.

The third sample is from the healthy control candidates collected by Gu et al. (2022). The subjects had no history of medical, neurological, or psychiatric disease. None of the subjects was taking medication at the time of testing.

**FIGURE 1:**
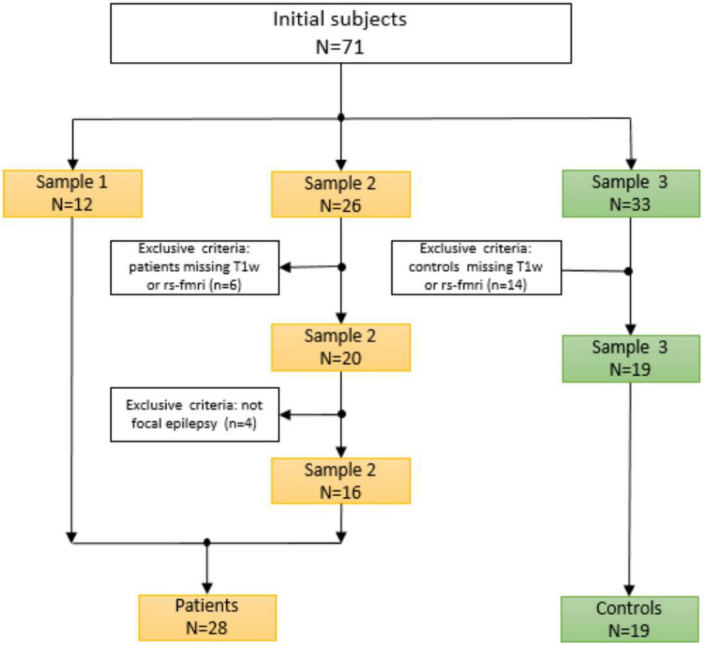
Criteria and the procedure for selecting subjects.

The image quality of all subjects and datasets was checked and controlled in a concise manner. The procedure and selection criteria were given in Figure 1. Our current study included a total of 47 subjects, including 28 patients and 19 healthy controls. All patients were collected and evaluated in accordance with standard principles. The evaluation included a detailed clinical history, a neurological examination, a complete neuropsychological assessment, a psychiatric assessment, and inter-ictal and ictal onset patterns in a long-term scalp video-electroencephalogram (video-EEG), MRI, and fMRI. They were all diagnosed with focal epilepsy, and the presurgical evaluation test results for each patient are shown in Table 1.

**TABLE 1:** Seizure onset zones for patients determined through the presurgical evaluation.

Patient	Seizure onset zone
01	Left frontal
02	Left middle temporal
03	Left frontal
04	Left middle temporal
05	Left middle temporal
06	Left middle temporal
07	Left middle temporal
08	Left middle and lateral temporal
09	Right hippocampus and temporal lobe
10	Left temporal lobe
11	Left hippocampus and middle temporal
12	Right frontal
13	Left mesial temporal lobe
14	Right mesial temporal lobe
15	Right hippocampus
16	Left occipital lobe
17	Left frontal cystic mass
18	Left mesial temporal lobe
19	Left frontal encephalomalacia
20	Right mesial temporal lobe
21	Right anterior frontal lobe
22	Left mesial temporal lobe
23	Left mesial temporal lobe and frontal lobe
24	Left temporal pole
25	Right mesial temporal lobe
26	Right mesial temporal lobe and right frontal pole
27	Right mesial temporal lobe, Possible right frontal base
28	Left mesial temporal lobe

This study was approved by the ethics committee of Shengjing Hospital of China Medical University and the informed consent was signed by the participant or a legal guardian/next of kin (for the participant under the age of 18).

### 2.2. Data acquisition

All rs-fMRI measurements were acquired and processed with a specific epilepsy protocol as used in the clinical routine. For sample 1, the MR images were acquired with a PET/MR scanner (SIGNA PET/MR; GE Healthcare, Waukesha, WI, USA) using a 16-channel head coil. The protocol included the following sequences: Sag 3D T1BRAVO (T1w; TR = 8.5 ms, TE = 3.3 ms, flip angle = 12°, voxel size = 0.469 × 0.469 × 1,000 mm^3^, FOV = 512 × 512). Resting-state BOLD images were acquired using a SIGNA PET/MR (TR = 2,000 ms, TE = 35 ms, Flip angle = 90 degrees, 3.5 × 3.4 × 4.0 mm^3^ voxel size).

Sample 2 images were acquired as follows: T1-W structural scans were obtained on a 3T GE Discovery 750w (BRAVO, 32 ch head coil, TE = 3.376 ms, TR = 8.588 ms, Flip angle = 12 deg., 1.0 × 1.0 × 0.8 mm voxel size). Resting-state BOLD-fMRI sessions were obtained in a subset of subjects before implantation (4.8 min per session, 32 ch head coil, TR = 2,260 ms, TE = 30 ms, Flip angle = 80 degrees, 3.4 × 3.4 × 4.0 mm^3^ voxel size).

Sample 3 data were collected on a 3 Tesla Prisma Siemens Fit scanner using a Siemens 20-channel receive-array coil. Anatomical images were acquired using an MPRAGE sequence (TR: 2,300 milliseconds, TE: 2.28 milliseconds, 1 mm isotropic spatial resolution, FOV: 256 millimeters, flip angle: 8 degrees, matrix size: 256 × 256 × 192, acceleration factor: 2). Each scanning section consisted of an anatomical session, two 10-min resting-state sessions, and several 15-min sleep sessions. Blood oxygenation level-dependent (BOLD) fMRI data were acquired using an EPI sequence (TR: 2,100 milliseconds, TE: 25 milliseconds, slice thickness: 4 mm, slices: 35, FOV: 240 mm, in-plane resolution: 3 mm × 3 mm).

### 2.3. Data processing

**FIGURE 2:**
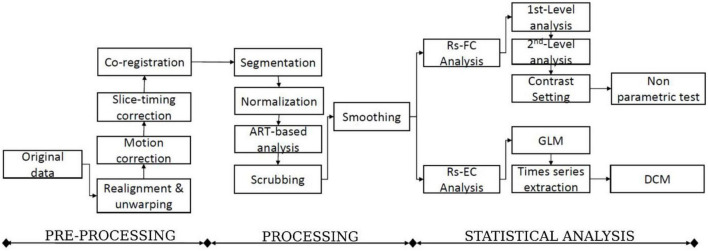
The flowchart of the functional and effective connectivity study (FC: Functional connectivity, EC: Effective connectivity, and DCM: Dynamic causal modeling).

The functional connectivity analysis in this study uses the CONN toolbox.^2^ CONN is a powerful and well-known neuroimaging toolbox that helps process task-related and rs-fMRI data (Whitfield-Gabrieli and Nieto-Castanon, 2012; Schurz et al., 2014). For this study, we subdivided our method into three main steps: data preprocessing, processing, and statistical analysis (Figure 2). The classical CONN pre-processing procedure was performed using the default configuration. It included the realignment and unwarping of the functional images, motion correction, slice-timing correction, and co-registration with the structural data (target resolution for functional images = 2 mm). Structural segmentation and normalization, functional normalization, ART-based (Artifact Detection Tools) functional outlier detection and scrubbing, and functional smoothing (full-width-at-half maximum [FWHM 8-mm Gaussian kernel) were carried out in MNI-space. After pre-processing data, the processing step will follow. The CONN default processing was set as it implements the component-based noise correction method (CompCor) strategy for physiological and other noise source reduction, additional removal of movement, and temporal covariates, temporal filtering, and windowing of the residual blood oxygen level-dependent (BOLD) contrast signal. During the processing step, resting-state signals will be extracted from the gray matter, and the cortex will be divided into different regions of interest (ROI). Additionally, the mean time series of each ROI will be extracted as regressors, where other internal processing conditions will be specified along with the regressors. After that, individual connectivity maps will be created for each participant. Eight nodes derived from the networks were selected as the atlases or regions of interest. One can refer to Table 2 for the details concerning the networks and coordinate information of their nodes.

**TABLE 2:** Network nodes and their coordinates.

Network	Node	Coordinate (*x*, *y*, *z*)
Default mode network	Medial prefrontal cortex (MPFC)	1, 55, −3
	Left lateral inferior parietal (LIPC)	−39, −77, 33
	Right lateral inferior parietal (RIPC)	47, −67, 29
	Posterior cingulate cortex (PCC)	1, −61, 38
Dorsal attention network	Left frontal eye field (L-FEF)	−27, −9, 64
	Right frontal eye field (R-FEF)	30, −6, 64
	Intraparietal sulcus (L-IPS)	−39, −43, 52
	Intraparietal sulcus (R-IPS)	39, −42, 54

### 2.4. Functional connectivity

The functional connectivity analysis includes the first-level and second-level analyses (Figure 2). The whole analysis was based on the seed-based connectivity (SBC) procedures defined in CONN tool. In the first-level analysis, for each subject, every region in DMN and DAN is used as a seed to conduct a seed-to-voxel functional connectivity analysis, such as to compute the correlation maps between the seed and voxels in the rest of the brain. DMN has 4 regions, including the posterior cingulate cortex (PCC), medial prefrontal cortex (MPFC), and bilateral inferior parietal cortex (LIPC and RIPC). DAN has four regions, including the frontal eye field (FEF) and the intraparietal sulcus (IPS) at the left and right hemispheres. Finally, a z-transformed connectivity map is obtained for each region of each subject.

In the second-level analysis, we first set a contrast (a label of “1” is given to a drug-resistant patient, and “0” is given to a healthy control) and highlight the functional connectivity for the group-level analysis. For each region in DMN and DAN, a non-parametric test was used to compare the z-transformed connectivity maps of each drug-resistant patient and the healthy control group. This means that the comparison will be performed 224 (28 patients by 8 regions) times. The obtained results were considered significant at a threshold of voxel-wise *p* < 0.001 uncorrected and cluster-level *p* < 0.05, false discovery rate (FDR) corrected for between-group comparisons. If one or more significant clusters are found for each patient, we consider significant connectivity changes.

Moreover, ROI-to-ROI connectivity analysis was performed to estimate the functional connectivity values between each pair of regions in DMN and DAN. The mean connectivity values of each region within each network were extracted for the patient and healthy control groups.

### 2.5. Effective connectivity

This study used the spectral dynamic causal modeling (DCM) approach to determine causal connectivity. All DCM analysis was performed with SPM12 according to the steps described in Sharaev et al. (2016). Spectral DCM analysis uses a neuronally plausible power-law model of the coupled dynamics of neuronal populations to generate complex cross spectra among the measured responses. Figure 1 shows the procedure included running general linear modeling followed by time series extraction, after which the DCM can be specified for each subject. The data used for the time series extraction includes data fully preprocessed during CONN analyses. After the GLM estimation, time series extraction was unsuccessful for 6 subjects, and we finally had 22 subjects for our further analysis.

**FIGURE 3:**
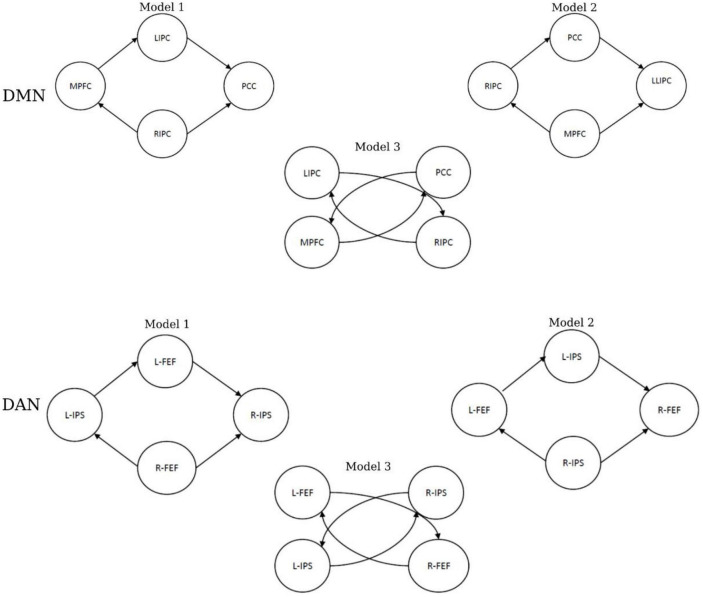
The specified models of effective connectivity in the default mode network (DMN) and dorsal attention network (DAN).

The challenge of the effective connectivity analysis was to assess the activities between different brain regions for two rs-fMRI networks. To achieve this goal, neural modeling schemes must be specified, and this will help in making inferences as it provides details about the interactivities and the connectivity strength between different regions (nodes) within a specific network (Friston et al., 2014; Bidhan and Mukesh, 2015). Therefore, six models were constructed for each subject. Figure 3 shows three models that were specified for the DMN and DAN. In addition, fixed effects (FFX) Bayesian Model Selection (BMS) was conducted to determine the best model that balances the data fitting and model convolution (Rosa et al., 2010). Moreover, for the best model, Bayesian Model Averaging (BMA) was conducted (Hinne et al., 2020). The probability-weighted values obtained from the BMA parameters models were quantitively analyzed using a classical one-sample *t*-test to examine the significance of the non-zero values.

### 2.6. Statistical analysis

After performing the functional and effective connectivity analysis, a statistical test was performed to evaluate our study’s results. All the statistical tests were performed using SPSS.

Once the average connectivity values among the pairs of each region were estimated in DMN and the DAN, these values were subjected to a two-sample *t*-test to verify the difference in connectivity between the patient group and healthy controls.

## 3. Results

### 3.1. Resting-state functional connectivity

Selecting the DMN and DAN networks as regional seeds, the functional connectivity analysis revealed drug-resistant epilepsy showing abnormal clusters in patients. For DMN, significant connectivity changes were observed in 64.3% (18/28) of patients. In comparison, 78.6% (22/28) of connectivity changes were observed for DAN.

**FIGURE 4:**
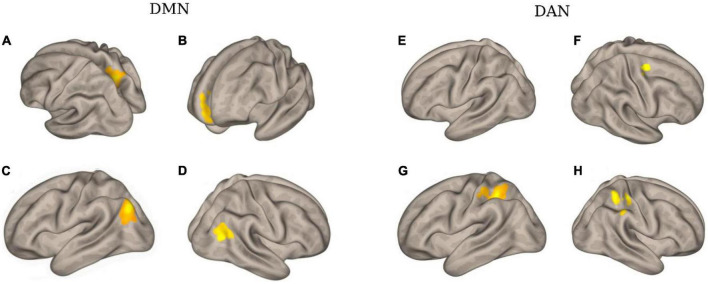
The clusters with different functional connectivity connected to the regions [as the seed) in the DMN and DAN network [here are the results of one patient (No. 02) as an example]. **(A)** Using PCC as the seed; **(B)** using MPFC as the seed; **(C)** using LIPC as the seed; **(D)** using RIPC as the seed; **(E)** using L-FEF as the seed; **(F)** using R-FEF as the seed; **(G)** using L-IPS as the seed; and **(H)** using R-IPS as the seed.

Figure 4 and Table 3 illustrate an example of the observed changes within the DMN and DAN for a single patient. The abnormal locations included the lateral occipital, middle, and superior temporal gyrus, medial frontal cortex, and superior parietal lobule for DMN. Whereas, for DAN, these changes were seen in the precentral gyrus, superior parietal lobule, inferior temporal gyrus, inferior frontal gyrus, and temporal fusiform cortex. Comparing the individual performance of each network, the left lateral parietal lobule and posterior cingulate cortex nodes had the highest number of patients with significant connectivity changes, with 42.4% (13/28) for DMN. The best connectivity performance for the DAN, on the other hand, was observed in the left intraparietal sulcus node, yielding 53.6% (15/28). Table 4 contains additional information about the findings of individual patient for both networks.

**TABLE 3:** An example of functional connectivity between one patient (No. 02) and healthy controls.

Network	Node (seed)	Region with clusters	Peak location (x, y, z)			*T*-value	The number of voxels
DMN	LIPC	L-LOC	−34	−78	36	7.96	681
	RIPC	R-LOC	48	−62	26	5.94	421
	MPFC	MedFC	0	58	−2	7.86	671
	PCC	Precu	6	−56	32	4.77	2,030
DAN	L-FEF	–	–	–	–	–	–
	R-FEF	R-PreCG	28	−6	34	5.04	54
	L-IPS	L-SPL	−34	−48	44	7.13	912
	R-IPS	R-SPL	34	−44	52	6.72	805

L, left; R, right; IPC, lateral parietal; MPFC, medial prefrontal cortex; IPC, inferior parietal cortex; PCC, posterior cingulate cortex; FEF, frontal eye field; IPS, intraparietal sulcus; LOC, lateral occipital; MedFC, frontal medial cortex; Precu, precuneous cortex; FP, frontal pole; SPL, superior parietal lobule; PreCG, precentral gyrus.

**TABLE 4:** The region with different functional connectivity to the regional seeds in DMN and DAN networks (obtained by comparing the generated functional connectivity mapping between each individual patient and the healthy control group).

Sub.	DMN				DAN			
	LIPC	RIPC	MPFC	PCC	L-FEF	R-FEF	L-IPS	R-IPS
		–	–	–	–	–	–	–
02	L-LOC	R-LOC	MedFC	Precu	–	R-PreCG	L-SPL	R-SPL
03	L-Caudate	L- tri IFG	–	R-MTG	L-FP	R-ITG	PC	R-STG
04	L-LOC	R-LOC	MedFC	Precu	–	R-PreCG	L-SPL	R-SPL
05	L-LG	L-LG	–	L-LG	L-LG	L-LG	L-LG	L-LG
06	L-LOC	R-LOC	MedFC	Precu	–	R-PreCG	L-SPL	R-SPL
07	-	R-LOC	MedFC	Precu	–	R-PreCG	L-SPL	R-SPL
08	–	–	–	–	–	–	–	–
09	L-oper IFG	L-AG	R-SPL	L-oper IFG	–	–	L-ITG	R-SPL
10	R-LOC	L-LOC	R-LOC	L-LOC	–	MedFC	–	–
11	R-OP	L-ITG	R-OP	L-OP	L-TFusC	L-TFusC	L-MFG	L-FG oper
12	L-LOC	L-LOC	MedFC	Precu	–	R-PreCG	R-SPL	R-SPL
13	L-LOC	–	–	–	–	–	R-ITG	L-LOC
14	–	–	–	–	–	–	–	R-SPL
15	–	–	–	–	–	–	R-SPL	–
16	–	–	–	–	R-MTG	L-OP	L-PostCG	–
17	–	R-Cereb	–	–	L-FP	–	–	–
18	–	–	–	–	–	–	L-SPL	–
19	–	–	R-Cereb	–	R-Cereb	–	L-SPL	–
20	–	–	–	–	–	–	–	–
21	–	–	FP l	–	L-PreCG	–	–	–
22	R-Cereb	R-Cereb	R-Cereb	R-Cereb	R-Cereb	R-Cereb	R-Cereb	L-FP
23	–	–	R-Cereb	–	R-Cereb	–	–	–
24	–	–	R-Cereb	–	R-SPL	–	–	–
25	–	–	Brain-S	Brain-S	Brain-S	Brain-S	R-LOC	Brain-S
26	R-Cereb	R-Cereb	R-Cereb	R-Cereb	R-Cereb	R-Cereb	R-Cereb	R-Cereb
27	–	–	–	–	L-PreCG	–	–	–
28	R-SPL	–	R-PreCG	-	R-Cereb	L-MTG	L-MTG	L-MTG

(-), no findings; L, left; R, right; LP, lateral parietal; MPFC, medial prefrontal cortex; PCC, posterior cingulate cortex; FEF, frontal eye field; IPS, intraparietal sulcus; OP, occipital lobe; LOC, lateral occipital; IFG, inferior frontal gyrus; MedFC, frontal medial cortex; Precu, precuneous cortex; FP, frontal pole; SPL, superior parietal lobule; PreCG, precentral gyrus; PostCG, post cingulate gyrus; ITG, inferior temporal gyrus; MTG, middle frontal gyrus; Cereb, cerebellum; Brain-S, brain stem; LG, lingual gyrus; AG, angular gyrus; oper, operculum; TFusC, temporal fusiform cortex.

**FIGURE 5:**
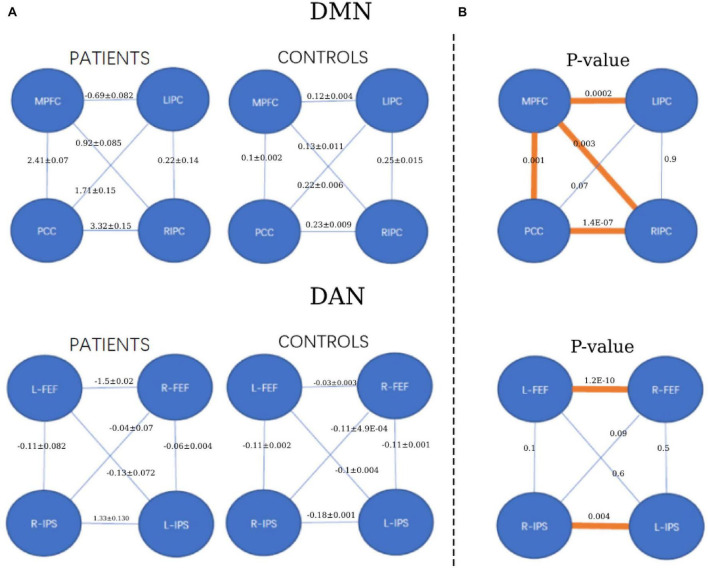
The strength of functional connectivity (mean and standard deviation) in the patient and healthy control groups and their comparisons. **(A)** The strength of FC for each group (patients and controls). **(B)** The *p*-value of FC comparisons between the patient and healthy control groups. *p < 0.05, orange colors.

Furthermore, the statistical analysis between the connectivity of patient and healthy control groups is shown in Figure 5. For the DMN, the two-sample *t*-test was significant between the medial prefrontal cortex and posterior cingulate cortex (*p* = 0.001) and the bilateral inferior parietal cortex (*p* = 0.0002 and 0.003). Significant connectivity was also found between the posterior cingulate cortex and the right inferior parietal cortex (*p* = 1.4e-07). For DAN, the two-sample *t*-test was significant between the left and right frontal eye fields (*p* = 1.2e-10). Additionally, significance was found between the left and right intraparietal sulcus (*p* = 0.004).

### 3.2. Resting-state effective connectivity

**FIGURE 6:**
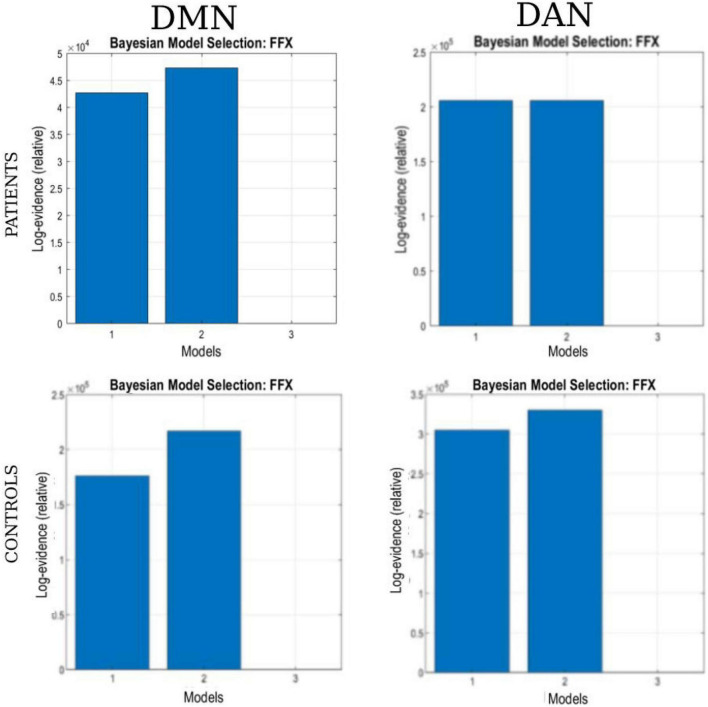
The Bayesian model selection (BMS) at the single-group level. The columns represent the network (DAN and DMN), and each row represents the study group (patients and healthy controls).

Bayesian model selection (BMS) for the patient and healthy control groups is shown in Figure 6. During BMS analysis, for the DMN and DAN, the fully connected models were the best for 4 of the 6 models specified for the patient and healthy control groups. For both networks, models 1 and 2 were the best for patients and healthy controls. At the group level, models 1 and 2 were the best for both networks in 22 out of 22 patients (90.9%). A similar scenario was observed for the healthy control group. Models 1 and 2 were the best for both networks in 17 out of 19 (89.5%) patients. For both networks, model 3 was weaker for the patient and healthy control groups. For the patient group, model 3 was better, with 3/22 (13.6%) and 4/22 (18.2%) for DMN and DAN, respectively. For the healthy control group, model 3 was the best for 2 out of 19 subjects (10.5%) for DMN and DAN.

The results of BMA and the *t*-test are shown in Tables 5A, B. The one-sample *t*-test analysis found different significant connections for each network and group. For DMN, the patient group yielded significant connectivity from the right inferior parietal cortex to the medial prefrontal cortex (EC = 0.02), bilateral connectivity was found between the medial prefrontal cortex and the posterior cingulate cortex (EC = 0.02), and bilateral connectivity in the inferior parietal cortex (EC = 0.02). Healthy controls did not show significant connectivity.

**TABLE 5:** The strength of effective connectivity (mean and standard deviation, in Hz) in the patient and healthy control groups and their comparisons.

(A)					
Group	Connection	From MPFC	From PCC	From LIPC	From RIPC
Patient	To MPFC	0	**0.02 ± 0.001**	0	**0.2 ± 0.003**
	To PCC	**0.02 ± 0.001**	0	**0.07 ± 0.005**	0.13 ± 0.002
	To LIPC	0.19 ± 0.004	0	0	**0.02 ± 0.001**
	To RIPC	0	0	**0.02 ± 0.001**	0
Healthy control	To MPFC	0	0	0	0.11 ± 0.001
	To PCC	0	0	0.07 ± 0.001	0.11 ± 0.001
	To LIPC	0.22 ± 0.003	0	0	0
	To RIPC	0	0	0	0
**(B)**					
**Group**	**Connection**	**From L-FEF**	**From R-FEF**	**From L-IPS**	**From R-IPS**
Patient	To L-FEF	0	0	**0.2 ± 0.003**	**0.03 ± 0.001**
	To R-FEF	0	0	0.01 ± 0.002	**0.01 ± 0.001**
	To L-IPS	**0.04 ± 0.002**	**0.2 ± 0.004**	0	0
	To R-IPS	0.13 ± 0.006	**0.13 ± 0.003**	0	0
Healthy control	To L-FEF	0	0	0.017 ± 0.001	0.15 ± 0.001
	To R-FEF	0	0	**0.04 ± 0.003**	0.13 ± 0.002
	To L-IPS	**0.15 ± 0.002**	**0.02 ± 0.0**	–0	0
	To R-IPS	−0.002 ± 0.001	−0.003 ± 0.0	0	0
**(C)**					
**Connection**		**From MPFC**	**From PCC**	**From LIPC**	**From RIPC**
To MPFC		0	0.7	0	0.6
To PCC		0.7	0	0	0.07
To LIPC		**5.50E-05**	0	0	**0.05**
To RIPC		0	0	0.7	0
**(D)**					
**Connection**		**From L-FEF**	**From R-FEF**	**From L-IPS**	**From R-IPS**
To L-FEF		0	0	0.9	**2.20E-14**
To R-FEF		0	0	**3.50E-07**	**7.70E-11**
To L-IPS		**1.30E-10**	0.8	0	0
To R-IPS		0.7	0.8	0	0

(A) Default mode network; (B) dorsal attention network; (C.) the p-value of comparison of EC between the patient and healthy control groups in DMN; (D) the p-value of comparison of EC between the patient and healthy control groups in DAN.

(A) The bold font represents the parameters with significant non-zero values by a one-sample t-test (p < 0.05).

(B) The bold font represents the parameters with significant non-zero values by a one-sample t-test (p < 0.05).

(C) The bold font represents significant differences in a two-sample t-test (p < 0.05).

(D) The bold font represents significant differences in a two-sample t-test (p < 0.05).

For DAN, the patient group yielded significant connectivity from the right frontal eye field to the left intraparietal sulcus (EC = 0.2) and from the right intraparietal sulcus to the left frontal eye field (EC = 0.03). Bilateral connectivity was found between the left frontal eye field and the left intraparietal sulcus (EC = 0.04, EC = 0.2), as well as between the right frontal eye field and the right intraparietal sulcus (EC = 0.13, EC = 0.03).

**FIGURE 7:**
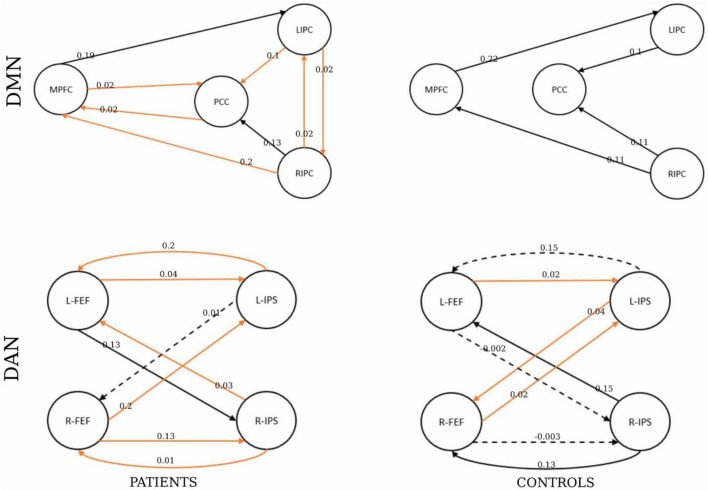
The winning model at the group level. The number shows the connectivity parameters (Hz) of the winning model in the patients and healthy control groups represented by the columns. The rows represent the network type. The solid lines represent connectivity values greater than 0.1 Hz, and their thickness shows the size of the value. The dotted lines represent the connectivity values below 0.1 Hz. The orange represents the parameters with significant non-zero values by a one-sample *t*-test (*p* < 0.05).

For the healthy control group, significant connectivity was observed from the left frontal eye field to the left intraparietal sulcus (EC = 0.15), from the right frontal eye field to the left intraparietal sulcus (EC = 0.02), and from the left intraparietal sulcus to the right frontal eye field (EC = 0.04). Non-trivial connections have been considered, and self-connections in graphs have been ignored for simplicity. In addition, only significant connections with a connection strength greater than 0.1 Hz and a probability greater than 0.95 were reported. The winning model that summarizes the strength of interactions within the networks for each group is shown in Figure 7.

Finally, the statistical analysis results of the effective connectivity values between the patient and healthy control groups are shown in Tables 5C, D. For the DMN, the two-sample *t*-test was significant from the middle prefrontal cortex to the left inferior parietal (*p* = 5.5E-05), and from the right inferior parietal cortex (*p* = 7.93E-09).

For the DAN, the two-sample *t*-test was significant from the left frontal eye field to the intraparietal sulcus (*p* = 1.30E-10), the left intraparietal sulcus to the right frontal eye field (*p* = 3.50E-07), and the right intraparietal sulcus to the left and left frontal eye fields (*p* = 2.20E-14 and 7.70E-11, respectively).

## 4. Discussion

### 4.1. Significance and importance of this study

Drug-resistant epilepsy remains one of the most severe cerebral diseases, and its diagnosis remains very tedious. Resting-state fMRI is an essential neuroimaging tool that has shown interesting results in diagnosing brain diseases. The purpose of this study is to investigate brain activities in rs-fMRI networks. The primary experiment of this study provides new information in the study of the connectivity of rs-fMRI networks of patients with drug-resistant epilepsy. Functional and effective connectivity analysis approaches were used to assess connectivity behaviors in the default mode and the dorsal attention networks. Overall, the study provides satisfactory results.

The FC analysis showed abnormal activities in 18 out of 28 patients for the DMN compared with the DAN, which showed slightly higher performance showing activities in 22 out of 28 patients. This result supports that DMN and DAN are two crucial rs-fMRI networks to be evaluated during the presurgical analysis of candidates for refractory epilepsy (Blumenfeld et al., 2004; Hinne et al., 2020). This assertion is confirmed by Widjaja et al. (2013), who reported a decreased FC within the DMN in children with medically refractory epilepsy. Zhou et al. (2020) also highlighted the crucial role of the DAN network by investigating right temporal lobe epilepsy candidates.

The DCM analysis performed well and showed connectivity for the DMN and DAN networks. The BMA values and the one-sample *t*-test analysis revealed significant connectivity within patient and healthy control group networks. For DMN, the EC was significant in the posterior cingulate cortex and medial prefrontal cortex for patients, while none was significant for healthy controls. This result suggests that the posterior cingulate and the middle prefrontal cortex represent two regions with high sensitivity for drug-resistant candidates. In their study, Cook et al. (2019) confirmed this observation in which the effective connectivity of the DMN in patients with left temporal lobe epilepsy was assessed using the spectral DCM approach. Their study revealed connections between the posterior cingulate and medial prefrontal cortex (Cook et al., 2019).

In addition, the patient group showed significant connectivity from the right intraparietal sulcus and all other nodes for DAN. Left and right bilateral connectivity was also observed in the intraparietal sulcus. This result suggests that the right parietal lobule is a sensitive region in the DAN. Zhou et al. (2020) examined cognitive damage of the DAN in patients with right temporal lobe epilepsy (rTLE) and found a significant difference in the right superior parietal lobule (SPL) and right precuneus (PCU) in patients with rTLE compared with healthy controls.

Furthermore, comparing the results of the DCM analysis of the group of patients to that of healthy controls, a significant difference in connectivity was also observed for the two networks. More activities were observed in the patient group compared with the control group. This hyper-activity observed within the group of patients may result from the frequent appearance of seizures in drug-resistant candidates. This last hypothesis (that abnormal interactions may cause frequent seizures in candidates for drug-resistant epilepsy) answers the objective of this study. Thus, the study provides evidence that effective connectivity is a powerful presurgical and post-surgical analysis technique. This analytical approach was used by Jiang et al. (2018). In their study, the Granger causality effective connectivity analysis approach was used to study the attention networks and default mode network of refractory participants. The specific disrupted networks appear to be associated with the specific cognitive characteristics of drug-resistant.

Finally, for the DMN, the statistical analysis of the FC showed significant differences between the medial prefrontal cortex and the posterior cingulate cortex and also between the posterior cingulate cortex and the right inferior parietal cortex. It was significant for DAN between the left and right frontal eye field and the intraparietal sulcus.

Moreover, for the DMN, the statistical analysis of the EC showed a significant difference from the right inferior parietal cortex to the middle prefrontal and posterior cingulate cortex. Additionally, the difference was observed from the posterior cingulate cortex to the middle prefrontal cortex and the left inferior parietal cortex. This result suggests that the brain networks of the patient and control groups exhibit different characteristics. Xiao et al. (2020) evaluated functional connectivity and topological properties of brain networks and found that their alterations were associated with neuropsychological disease.

### 4.2. Importance of resting state in drug-resistant epilepsy analysis

Our study proposes to assess the functional and effective connectivity of the default mode and the dorsal attention networks, two networks known to present altered connectivity for epilepsy patients. The vital role of rs-fMRI in assessing altered brain connectivity for epilepsy patients has been investigated by several researchers.

These assessments have demonstrated noticeable progress. Boerwinkle et al. (2020) prospectively examined the influence of rs-fMRI on the organization of pediatric epilepsy surgery. Jiang et al. (2018) also assessed the functional and causal connectivity of the attention networks and default mode network using rs-fMRI and revealed that epileptic activity might disrupt network interactions and further influence information communication. Zhang et al. (2011) also investigated epilepsy networks using resting-state fMRI, emphasized the importance of local network topology when investigating mechanisms underlying tumor-related epilepsy, and provided motivation for further investigation of the epilepsy process at the network level.

### 4.3. Functional and effective connectivity performance comparison

Brain connectivity is defined as a pattern of interactions between the different areas of the brain. Functional connectivity focuses on the temporal correlation among the activity of different brain areas, while effective connectivity relies on the causal interactions among the activity of different brain areas. The fundamental difference between functional and effective connectivity is the temporal implication of the source of the effect, and this study has investigated the importance of functional and effectivity analysis for the presurgical analysis of drug-resistant. We also tried to compare the best approach to be used when trying to investigate rs-fMRI patients. However, our evaluation revealed that both connectivity methods could answer our needs at different levels. The descriptions above answer this study’s goal, combining both methods to bring out meaningful answers and facilitate surgical operations for drug-resistant candidates.

Several researchers who used and compared both analyses approaches confirmed this study’s assumption. Among them, Saetia et al. (2020) studied the interpretability of the effective connectivity model compared with the functional connectivity model. Park et al. (2018) also evaluated the ensuing dynamic effective connectivity in terms of the consistency of baseline connectivity within DMN using the rs-fMRI. They speculated that human brain networks at rest show dynamic functional connectivity induced by effective dynamic connectivity, which can be modeled efficiently using dynamic causal modeling and hierarchical Bayesian inference. Mao et al. (2020) investigated the functional connectivity and effective connectivity of the habenula in 34 subjects with irritable bowel syndrome (IBS) and 34 healthy controls and assessed the feasibility of differentiating IBS patients from healthy controls using a machine learning method.

### 4.4. Limitations and challenges

Despite the promising results obtained during our study, some challenges and limitations to this approach persist and must be overcome. First, a seed-based analysis is known as a relatively assumption-based approach. It requires the *a priori* selection of a specific voxel, atlas, or network. However, the choice of seed may biologically bias the connectivity findings toward specific, smaller, or overlapping sub-systems rather than larger, distinct networks (Cole et al., 2010). The combination of a data and hypothesis-driven approach may provide a suitable answer to this problem (McKeown, 2000; Caulo et al., 2011). Second, the data size is crucial during analysis because a small sample size limits the statistical power. The sample size in this study is relatively small, which may impact the results (Grady et al., 2021). This issue must be considered preliminary and needs to be replicated in future studies with larger sample sizes and more detailed scale tests. Third, the lack of post-operative outcomes information in both patient samples makes comparison with our findings impossible. Fourth, different acquisition procedures may have an adverse effect on the interpretability of the result. The rs-fMRI population used in this study was acquired using slightly differential procedures. For the patients, sample 1 was obtained with the eyes closed, while sample 2 was obtained with the eyes open. For the healthy control group, a mixed eyes open-closed acquisition was used. This obvious acquisition difference may have an effect on each candidate’s brain activity in sensorimotor and occipital regions (Wei et al., 2018). This limitation, however, has not resulted in statistical evidence and only a small portion of brain activity is affected (Agcaoglu et al., 2019). This claim is supported by Patriat et al. (2013), who discovered that when the acquisition procedure was changed, only the visual network changed significantly.

Finally we also explored node-based connectivity analysis using the DCM method in this study. The expressiveness or complexity of the underlying neural model limits the interpretability of DCM. This complexity is constrained by the nature of the data at hand (Sadeghi et al., 2020). The strengths of the DCM approach lie in the hemodynamic model that links neuronal population firing to BOLD data, which creates potential mismatches. This mismatch may result in incorrect edge strength estimates within the DCM, potentially leading to the selection of wrong edge configurations. Another limitation concerns the large number of nodes used in the resting state analysis, leading to a considerable number of parameters in the DCM, making estimation difficult. Perhaps anatomical connectivity analysis can help reduce some of the challenges.

## 5. Conclusion

Brain connectivity analysis has always been challenging for researchers. This study characterized drug-resistant epilepsy by assessing functional and effective connectivity within resting state networks. The DMN and DAN networks were investigated at a subject and group level. Our analysis provided evidence of abnormal functional connectivity for the DMN and DAN. In addition, dynamic causal modeling analysis has shown significant effective connectivity within both networks. Finally, the statistical analysis has demonstrated the connectivity differences within the networks of both patients and healthy control groups. Our findings provide preliminary evidence to support that combining functional and effective connectivity analysis may highly contribute to diagnosing altered brain networks in drug-resistant candidates. The results of this research may offer new insight into the neuropathophysiological mechanisms of brain network dysfunction in drug-resistant epilepsy. In our subsequent studies, we will examine connectivity patterns between the DAN and DMN.

## Data availability statement

The datasets presented in this article are not readily available because of the requirement of the Ethics Committee of Shengjing Hospital of China Medical University (Shenyang, China). Requests to access the datasets should be directed to HL, leoincmu@gmail.com.

## Ethics statement

The studies involving human participants were reviewed and approved by the Ethics Committee of Shengjing Hospital of China Medical University. Written informed consent to participate in this study was provided by the participants’ legal guardian/next of kin.

## Author contributions

EB performed experiments and analyzed the data along with SQ and CJ. SQ, DH, and HL conceived the study, presented the results, and wrote the manuscript along with EB. SH and LW collected and analyzed the data. SQ and LW supervised the algorithm development and analyzed the data. All authors read and approved the final manuscript.

## Footnotes

1
openneuro.org

2
www.nitrc:projects/conn
